# Book Review

**Published:** 1914-06

**Authors:** 


**Ionic Medication.** By H. LEWIS JONES, M.D. Pp. viii, 151. London: H. K. Lewis. 1913. Price 5s. net.—Those of our readers who remember Dr. Lewis Jones' excellent address on "Treatment by Cataphoresis," given a few years ago before the Bristol Medico-Chirurgical Society, will welcome the appearance of this little book. It contains a short discussion on the physics of ionisation, as well as complete directions for the administration of remedies by this means. The various kinds of ions available for therapeutic use are described in detail, and the maladies in which benefit may be expected are set forth at length. The book is essentially one to be recommended to the general practitioner. It will add materially to his resources in the treatment of painful and obstinate cases of many sorts.

**A Manual of Medical Treatment, or Clinical Therapeutics.** By I. BURNEY YEO, M.D. Fifth Edition. By RAYMOND CRAWFURD, M.A., M.D., and E. FARQUHAR BUZZARD, M.A., M.D. Two volumes. Pp. xv, 834; vii, 846. London: Cassell & Co. Ltd. 1913. Price 25s.—Previous editions of this work were reviewed in Vols xii, xx and xxvii. It has now undergone most careful revision and some little enlargement, necessitated by a notice of new therapeutic methods and an amplification of the index. The advances made in the domain of vaccine therapy, the use of the X-rays in diagnosis, and the serviceability of various physical methods of treatment have received careful consideration. Selections of formulæ are still added and mostly without abbreviation, as in these days of ready-made combinations and proprietary remedies such formulæ have a decided educational value. The sections on nerve diseases have been revised by Dr. Farquhar Buzzard, and all the remaining sections by Dr. Raymond Crawfurd. The new and revised form of the book must enable it to maintain and enhance that reputation which it has so deservedly obtained in the past.

**Physics.** By C. G. Knott, D.Sc., F.R.S.E. Third Edition. Pp. 292, 370. London: W. & R. Chambers Limited. 1913. Price 7s. 6d.—The author has revised and amplified the previous edition of this book in the light of recent research, introducing a chapter on radio-activity and bringing the electron theory more into prominence. This has naturally lead to an increase in size. The book in its present form is an admirable text-book for the student who intends to proceed to an advanced course in Physics, but one is inclined to think that the six hundred and forty odd pages which it contains embrace more of Physics than the average medical student in his first year may reasonably be expected to assimilate. The general arrangement is good, and most of the diagrams are clear, but exception must be taken to several in Chapter xi, notably those on page 150, which convey quite an erroneous idea of the character of electric discharge. There are tables of optical constants at the end, but the general character of the book would lead one to look for data of more use to the elementary student than values for the refractive indices of various substances to five or six figures. Answers are given to the numerical examples, and the book is well indexed.

**Weitere Beiträge zur Behandlung der Nephritis und verwandter Erscheinungen.** Von Martin H. Fischer. Cincinnati. **Sonderabdruck aus Kolloidchemische Beihefte.** Band IV. Pp. 70. Dresden and Leipzig: Verlag v. Theodor Steinkopff. 1913.—In former papers the author has endeavoured to show that the symptoms of nephritis are due to colloido-chemical changes, which result in an abnormal production and accumulation of acid in the kidneys. Albuminuria is attributed to solution of kidney-colloids, the presence of casts to solution of binding material between parenchymatous cells and connective tissue by the acid, and the enlargement and alteration in colour of the kidneys to the same cause. The general symptoms of nephritis, *e.g.* anuria, anasarca, œdema, retinal changes, convulsions, etc., are attributed to accumulations of acid in the affected organs, increasing the affinity for water of the tissue colloids. The so-called complications of nephritis are not consequences of the kidney disease, but evidence of the simultaneous presence in other organs of the same changes that in the kidney are called nephritis. The object of treatment is to combat the accumulation and over-production of acid. In mild cases this is done by the administration of hourly doses of sodium carbonate 0.5 to 1 grm. in water. If the patient cannot take this or does not speedily show improvement, rectal injections of sodium carbonate and chloride in distilled water are substituted; whilst in severe cases with urgent symptoms recourse is had to intravenous injection of the same salts in 1 to 2 litres of partly distilled water. The author gives a number of cases illustrating the (mostly) successful results of this treatment, which has also been successful in acute glaucoma.

**Guide to the Microscopic Examination of the Eye.** By Professor R. Greeff, with the co-operation of Professors Stock and Wintersteiner. Translated from the third German Edition by Hugh Walker, M.A., M.B., C.M. Pp. xvi, 86. London: The Ophthalmoscope Press. Price 7s. 6d. net. This is a conveniently-arranged handbook for those who are doing microscopical examination of eyes. There is a good index, and the methods are given clearly and in detail. A short account of the commoner bacteria is appended. The major part of the contents is to be found in the general books upon the subject, but there are a few "special" hints which justify the issue of another publication.

**Department of Neurology.** Harvard Medical School. Vol. V 1912. Boston, U.S.A.—This volume of reprints of papers by the neurologists of the Harvard Medical School, collected from various journals, maintains the high standard of its predecessors. We recommend its perusal to all students of neurology. The frontispiece is a portrait of Professor James Jackson Putnam, who retired from active service at the end of 1912, after being engaged in the teaching of neurology since 1872. Dr. Putnam has for years been one of the most distinguished members of the great neurological fraternity of the United States. That his activities have not ceased is shown by the fact that he contributes no less than eight papers to the present volume, dealing with the psychoneuroses and Freud's method of psycho-analysis. The volume contains many other papers of interest and value.

**A Guide to the Mental Deficiency Act, 1913.** By John Wormald and Samuel Wormald. Pp. x, 145. London: P. S. King & Son. 1913. Price 5s. net.—As this Act came into force on April 1st, 1914, and will revolutionise the status of the feeble-minded, both children and adults, it will be necessary for every medical practitioner to be conversant with its provisions. The Act is a far-reaching one, and should in time enable the problem of the feeble-minded, one of the most urgent and pressing social problems of our time, to be met. This Act is the first attempt to deal with the feeble-minded population in an adequate and scientific way, to prevent their unchecked increase, and to guard them from the commission of crime and from exploitation by evil-disposed persons. The book under notice comprises an introduction dealing with the preliminary stages of the passing of the Act of 1913, the authors' commentaries upon and explanations of its provisions, an appendix giving the full text of the measure, and an index. The explanations are given in a lucid manner, and the authors make numerous suggestions as to the way in which the powers given under the Act can be most fully utilised. We think that our readers will find this little work a useful guide, but with regard to the index, we would suggest that it should also give direct references to the Act itself, and not only as at present to the authors' explanatory text.

**Geburtshilfliches Vademekum für Studierende und Aerzte.** Von Prof. Dr. A. Dührssen. Zehnte auflage. Pp. xii, 308. Berlin: S. Karger. 1913.—The fact that this book has reached its tenth edition testifies to its popularity, and a perusal of it shows that this appreciation is well merited. The author's object of providing a vade mecum is kept in view throughout, and in the account of the physiology and pathology of labour stress is laid only on those points which are of practical importance, other matters of more theoretical interest being touched upon just enough to give the reader an intelligent view of the subject. In the section on treatment the teaching is clear and dogmatic, and embodies the methods practised in the author's clinic. In a book like the present one this is as it should be; for discussion and comparison of alternative methods the reader can go to larger text-books. We note that in the treatment of eclampsia the author strongly recommends emptying of the uterus, and praises but faintly the use of morphia for control of the fits. For placenta prævia he gives version the preference over the use of the Champetier de Ribes' bag, and in postpartum hemorrhage he is an advocate of early packing of the uterus, and not of keeping this proceeding as a last resource for extreme cases only. There is an excellent section on the causes of sudden death in connection with labour and the puerperium. The poorest parts of the book are the illustrations, which are scanty, and executed after the manner of text-books of years ago. That is a minor point, however, in a work of this kind, and in no way hinders its recommendation to students and practitioners as a most useful practical guide in the conduct of normal or difficult labour.

**Explanatory Lectures for Nurses and their Teachers.** By H. HAWKINS-DEMPSTER. Pp. xi, 224. Bristol: John Wright and Sons Ltd. 1913. Price 3s. 6d. net.—This volume consists of a series of lectures on nursing subjects, selected more or less at random. They are addressed to those in training for first aid certificates and as cottage nurses and the like. The subject-matter is very largely theoretical, and the style not very readable. The information given, however, is very accurate, although the statement that all micro-organisms are certainly killed by a temperature at freezing-point is open to criticism. However, the would-be cottage nurse can glean a great deal of useful information from the book, for which the price asked is certainly most reasonable.

**Cunningham's Manual of Practical Anatomy.** Revised and edited by ARTHUR ROBINSON. Sixth edition. Vol. I. Pp. xxx, 673. Edinburgh, Glasgow and London: Henry Frowde and Hodder & Stoughton. 1914. Price, 10s. 6d. net.—It is but eight years since the first edition of this well-known manual of anatomy appeared, and now the sixth edition is before us. Such a record speaks for itself, showing that the book has gained great popularity. In our opinion the popularity it enjoys is well deserved and will be maintained. For the present edition the whole of the text has been revised, new figures introduced, and a series of radiographs of joints and other organs appended.

**A Companion to Manuals of Practical Anatomy.** By E. B. JAMIESON, M.D. Pp. vii, 543. London: Henry Frowde and Hodder & Stoughton. 1913. Price, 6s. net.—This little book will be found useful to a student revising anatomy. Although of pocket size, by judicious condensing it contains all the essentials of the text-book in its 540 pages. It is also intended for use as a dissector's guide, an account being given of the relations in the more important regions, and, what we consider an important innovation, the various structures are dealt with under their systems, so that the student may realise that the structures engaging his attention are found also in other parts of the body. In fact, this work combines in one neat little volume the text-book and dissecting manual. It is matter for regret, however, that the Basle nomenclature has been adopted, although the older terms are in most cases inserted in brackets after the new. We hope also that in a future edition more attention will be given to surface-markings. Much credit is due to the author for the very careful editing to which he has subjected his work.

**The Early Diagnosis of Tubercle.** By CLIVE RIVIÈRE, M.D. Pp. x, 260. London: Oxford Medical Publications. 1914. Price, 6s. net.—We hold with the author that "the skill required for the early diagnosis of tubercle is, unfortunately, difficult and laborious to acquire, and no short cut to it does, or ever can, exist—the path of experience must be duly trod. The way can, nevertheless, be greatly smoothed by a careful piecing together of the scattered stones of knowledge over which the journey lies, and this has been attempted in the present book." It is divided into two sections: Part I—Adults, the diagnosis of pulmonary tuberculosis of adults, phthisis; and Part II—Children, tuberculosis of thoracic glands, hilus tuberculosis. The author starts with the statement, which cannot be denied, that "the first beginnings are generally overlooked, or are passed over as a febricula, or are labelled influenza, and subside again without arousing suspicion, hence it has nearly always reached a diagnosable stage by the time the physician is called in." The skill required for such early diagnosis is greatly dependent on certain special and laboratory tests which in themselves are often misleading; but we cannot dispense with the older methods of physical examination, and these must be aided by certain modern methods which require much time and prolonged training. But in nearly all our signs and tests the absolute is wanting, and the evidence from all sources has to be dealt with in a judicial spirit. The book presents fully the points whereby an accurate technique in the various methods may be acquired. Some 170 pages are occupied in the details of clinical diagnosis and the special diagnostic tests, and the student cannot fail to see how much he has to learn, and the older practitioner how much he has failed to learn, in the diagnosis of phthisis from this modern standpoint. As regards Part II, the author occupies some sixty pages on the clinical diagnosis and special diagnostic tests relating to hilus tuberculosis in children. There is much new matter here requiring careful study. A copious bibliography of over ten pages adds greatly to the value of the volume, on which we cannot do otherwise than congratulate the author, and of which he may well be proud. Without a careful study of this book no one can claim to be well versed in all the details of diagnosis of tuberculous diseases a subject in which modern workers have made such great advances during the last few years. The author has done a great deal towards the advancement of his speciality, and his work should do much towards the extinction of many fallacies and errors in the diagnosis of tuberculous diseases.

**Plain Rules for the Use of Tuberculin.** By R. ALLAN BENNETT, M.B.Lond. Pp. 48. Bristol: John Wright & Sons Ltd. 1914. Price, 2s. 6d. net.—The interest of this booklet is derived from the fact that it is written by one of our tuberculosis officers, in charge of the Devon County Council Tuberculosis

Dispensary, Torquay, and who was formerly R.M.O. to the Manchester Hospital for Consumption and Diseases of the Chest. It is intended as a guide to those who are working out for themselves the problems of tuberculin treatment. The author again emphasises the possibilities of errors in diagnosis of the disease, and of the so-called specific reactions as tests, and he goes on to illustrate the effects of tuberculin as it can be given to ordinary working-class patients at a dispensary. He contrasts the extravagant claims which are made for tuberculin from time to time with the views of those who hold that it is still on its trial in pulmonary tuberculosis, and he claims for it that it is one of the best weapons we have in the campaign against tuberculosis. He prefers the bovine tuberculin as a rule, and finds few cases which cannot be helped by its use. He appears to proceed with caution. He considers a temperature of 99.4 to be an absolute bar to its use, and he discontinues it in persons who are not capable of response. He holds that tuberculin treatment plus sanatorium must be immeasurably superior to tuberculin alone. These are views which commend themselves, and the book cannot fail to be a simple and reliable companion to the early worker on tuberculin in phthisis.

**A Synopsis of Surgery.** By Ernest W. Hey Groves, M.A., M.D., B.Sc. Lond., F.R.C.S. Eng. Fourth edition. Pp. x, 599. Bristol: John Wright & Sons Ltd., 1914. Price, 9s. 6d. net.—When a book reaches its fourth edition it is obvious that it meets with approval and has justified its existence. The present edition has been carefully revised, and sections on recent theories of shock, spinal cord tumours, resection of nerve roots and other subjects have been added. The most important new feature is the handing over of the pathological and bacteriological sections to Dr. George Scott Williamson, the Pathologist to the Bristol General Hospital. The information given is clear and accurate, and the most modern ideas have been incorporated. There is an excellent index, which is most helpful in a work of this kind. Cohnheim is spelt Conheim both in the index and body of the book.

**Index-Catalogue of the Library of the Surgeon-General's Office, United States Army.** Authors and subjects. Second Series, Vol. xviii. Pp. 1,057. "Tetamore-Tzschirner." Washington. 1913.—The volume includes 3,865 author titles, representing 4,117 volumes and 2,693 pamphlets. It also contains 5223 subject-titles of separate books and pamphlets, and 45,525 titles of articles in periodicals. These figures are so large, that it is better to refer to one or two headings in order to show more clearly the great utility of this work, which places the profession under a great debt to the collaborators. Under the heading "Tuberculosis" we have more than 110 pages of closely-printed references, the subject being divided under innumerable headings. More than sixty pages are similarly devoted to "Tumours"; thirty-five pages are occupied by references to thyroid glands and allied subjects, and thirty-eight to tetanus. The mention of these titles is sufficient to show how vast the literature has become, and how impossible it would be for writers on such subjects to deal efficiently with the matter without the help of such a praiseworthy and carefully-prepared work of reference.

**The Road to a Healthy Old Age. Essays Lay and Medical.** By Thos. Bodley Scott. Pp. 104. London: H. K. Lewis. 1914. Price 3s. 6d. net.—The title is a very captious one, and the author has no doubt contributed by his judicious treatments to the comfort and welfare of many of his patients, and has thereby justly earned their esteem and good will. But after careful perusal of the work, the question arises, do these sufferers from high tension and pulmonary and cardiac complaints ever live to very advanced ages? We have in our mind individuals (and could number many if space allowed) to whom high tension remedies are unknown by any real personal experience, and the only vaccine known that of infancy, and not of necessity even this. These people are very much a class to themselves, and by no means necessarily an hereditary one, for who can disentangle the skein of marriage? But even this class should not and cannot continue taking undue liberties with their constitution; they must live fairly straight and sober lives, and be active advocates for plenty of exercise, cold or tepid baths, and a mild system of gymnastics suited to their capabilities. We can speak with very little experience of the hippurates (p. 53–4), but we notice they have been given in combination with other heart tonics, and our own best results, with iodide of potassium, in arterio-sclerosis, have been with smaller doses, two, three, and rarely four gr. doses. Specialism is rather condemned on page 2, but rather exalted on page 84–5, where the author's son is being introduced on the stage of "vaccines."

**Dental Diseases in Relation to Public Health.** By J. Sim Wallace, D.Sc., M.D., L.D.S. Pp. viii, 90. London: *The Dental Record*, 1914. Price 3s. net.—A book that should be read by every medical practitioner and student. The advice about food and feeding is particularly good, and if followed would save an enormous amount of dental disease; but sufficient stress has not been laid on the use of the tooth brush and silk after meals.

